# Clinical Hypnosis in Medical Care: A Mixed-Method Feasibility Study

**DOI:** 10.1177/15347354211058678

**Published:** 2021-11-24

**Authors:** Sofie Bulling Lind, Henrik Børsting Jacobsen, Ole André Solbakken, Silje Endresen Reme

**Affiliations:** 1 University of Oslo, Oslo, Norway; 2 Oslo University Hospital, Oslo, Norway

**Keywords:** preoperative hypnosis, breast cancer surgery, postoperative pain, adverse side effects, integrative oncology

## Abstract

**Background:**

Preoperative hypnosis has shown promising effects in controlling side effects from breast cancer surgery, but the feasibility and effects are largely unknown outside the US.

**Methods:**

A mixed-methods approach was applied involving a large-scale population survey and a small-scale pilot study. The survey assessed attitudes toward hypnosis in a representative sample from the general population (n = 1049), while the pilot study involved interviews with 5 women who received hypnosis prior to mastectomy/lumpectomy.

**Results:**

In the survey, 8% reported to have previous experience with hypnosis, and 67% reported willingness to accept hypnosis in a medical setting. Increasing age was associated with more skepticism, while previous experience was associated with less skepticism. In the pilot study, 4 themes were identified: (1) caretaking, (2) experiences related to hypnosis, (3) thoughts and feelings related to diagnosis, and (4) surgery. All participants reported positive experiences related to hypnosis, and none described unpleasant side effects or postoperative pain (pain intensity > 3) after surgery.

**Conclusions:**

The results indicate that the general public is positive toward clinical hypnosis as a supplement to medical treatment and that preoperative hypnosis is feasible in Norwegian breast cancer patients.

**Trial Registration:**

ClinicalTrials.gov Identifier: NCT04300283.

## Introduction

Severe acute postsurgical pain is a common side effect from surgery. Apart from causing severe suffering in the acute phase, it is a well-documented risk factor for chronic postsurgical pain.^1^ The incidence of chronic postsurgical pain is high in several surgical populations, but particularly high after breast cancer surgery.^2^ Breast cancer is the most common type of cancer in women, with more than 3500 breast cancer surgeries performed every year in Norway^3^ and more than 1 million worldwide.^4^

Clinical hypnosis has received increasing attention the last 2 decades, with several studies documenting its effectiveness as a non-pharmacological method to reduce both the intensity and incidence of several postsurgical side effects, including pain.^5-7^ Hypnosis has been defined in various ways, but we conform to the definition by Montgomery et al^7^ who describe hypnosis as an agreement between the hypnotherapist and the patient to engage in a psychotherapeutic technique involving suggestions for changes in sensation, perception, cognition, affect, mood, or behavior. Although several psychological techniques can be helpful in a medical context, hypnosis appears to be particularly effective, and superior to other psychological techniques when it comes to preventing postsurgical side effects.^8^

Clinical hypnosis is not part of the usual treatment offered to women with breast cancer in Norway. To the best of our knowledge, no clinical studies have investigated the use of hypnosis as an adjunctive treatment in breast cancer surgery in Norway or any other Scandinavian country. Several studies have, however, been conducted in the US, with promising results.^5^ The lack of replication outside of the US context is nevertheless a limitation that calls for more studies. As a first step, we aimed to conduct a mixed-method feasibility study to prepare the ground for a larger trial in a Norwegian context of patients with breast cancer.

Several aspects are important to consider when planning for a randomized controlled trial in a new context. First, what are the attitudes toward the intervention in the general population? Any successful implementation of a preoperative hypnosis intervention will to a certain extent rely on public approval and willingness to accept hypnosis as part of medical care. This is particularly relevant in the context of a clinical trial where effects and potential side effects are partly unknown. If willingness is low in the general population, we risk a positively biased sample and slow recruitment. Second, the procedure needs to be tested in the clinical context, and the patients’ willingness to participate, as well as preliminary experiences with the intervention need to be investigated before proceeding further. The latter is also to detect any need for modification of the procedure and/or the intervention.

Although no studies of attitudes toward hypnosis have been conducted in a Scandinavian context, several studies have been done in the US. A recent review concludes that there is a generally positive attitude and openness toward hypnotherapy among the majority of people, but that hypnosis is less commonly viewed as effective for treating medical problems.^9^ We also know from previous studies that attitudes vary as a function of where people get their information, and whether they have previous experience with hypnosis. No previous knowledge about hypnosis, or knowledge from nonscientific sources, are associated with more negative attitudes and views, while previous experience with hypnosis—particularly when provided by a psychologist—has been associated with more positive attitudes and views.^10^ In a representative US survey, mostly positive or neutral views of hypnosis were detected, with 7.6% of the respondents reporting to have undergone hypnosis themselves.^11^

Whether these results could be replicated in a Norwegian population is still unknown. Krouwel et al^9^ claim that people’s perceptions about hypnosis are not so different across countries. Still, one might speculate that there are differences in the populations’ attitudes toward hypnosis due to cultural differences. Admittedly, the number of hospitals in Norway that offer complementary and alternative medicine (CAM) has increased steadily since the beginning of the 21st century, from 28% in 2001 to 64% in 2013.^12-14^ Although the definition of CAM also includes hypnosis, we do not know whether hypnosis has been offered at all, or to what extent patients are willing to accept hypnosis in a medical setting.

Moreover, few qualitative studies have investigated breast cancer patients’ experiences with hypnosis. One rare exception is an English study of 6 patients diagnosed with metastatic breast cancer reporting on their experiences with practices that included self-hypnosis. They all described that the interventions gave them a sense of control and empowerment, helping them to become active agents instead of passive receivers of treatment. Consistently, all the participants experienced that the intervention had helped them cope with their situation.^15^ The need for effective ways to cope with side effects from surgery, as well as other symptoms experienced by breast cancer patients, is evident from the literature on symptom experiences and quality of life in this patient group.^16-19^

The aim of this study was to assess the feasibility of preoperative hypnosis among women undergoing breast cancer surgery in Norway. We applied a mixed-method approach involving (1) a representative survey of the general population where experience with and willingness to accept hypnosis as part of medical care were assessed, and (2) a qualitative pilot study where the experiences of 5 women who received preoperative hypnosis were explored. Together, this will provide an indication of feasibility of a large-scale clinical trial, through securing information about acceptability, treatment response, recruitment capacity, and data collection procedures.^20,21^

## Methods

### The Survey

The attitudes toward hypnosis in Norway have, to our knowledge, never been investigated in a population-based sample. Therefore, in addition to the pilot study, we carried out a survey in collaboration with Ipsos, a commercial provider of polling data. We asked a representative sample of Norwegians the following 2 questions:

1. *Have you ever tried hypnosis?* (“Yes,” “No,” or “Do not know”).
2. *Hypnosis is sometimes useful in medical treatment. How likely is it that you would have said yes to hypnosis carried out by health care professionals?* (“Very likely,” “Pretty likely,” “A bit likely,” “Not likely at all,” or “Do not know”).

The survey was carried out between February 13th and February 27th, 2019. The sample for this study was randomly drawn from Ipsos’ online panel. Ipsos uses quotas on gender, age, and region, in drawing a sample to ensure a representative distribution. After a sample has been obtained from the Ipsos online panel, it is de-identified before Ipsos calibrates respondent characteristics to be representative of the Norwegian population using standard procedures, such as raking-ratio adjustments. The source of these population targets is census data. The sample drawn for this study reflects fixed sample targets on demographics. Post-hoc weights were made to the population characteristics on gender, age, and geography.

*Statistical analyses*: The data from the survey were analyzed using IBM SPSS version 27, through descriptive statistics (frequency and proportions) and a correlation matrix to show significant correlations between demographic characteristics and response to the hypnosis questions. Those responding “Do not know” on question 1 or 2 were excluded from the correlation analysis.

*Ethics and data protection*: Participation in the survey was voluntary and based on informed consent. The sample was drawn from Ipsos’ online panel of participants who had agreed to take surveys on a regular basis, and who consented to participate in the current survey including our questions. Participants in Ipsos panels receive points based on the length of the survey that could be redeemed in gift cards or small gifts. The data file we received were anonymized and could not be traced back to individual participants. In Norway, these kind of anonymous surveys are exempted from IRB review.

### The Pilot Study

Women scheduled for breast cancer surgery at Oslo University Hospital (OUH) between September 2018 and January 2019, were asked to participate in the pilot study. Five women agreed to participate, and they received adjunctive hypnosis prior to their surgery. Postoperatively, semi-structured interviews were conducted. These interviews were transcribed and analyzed qualitatively, with the intent of exploring the participants’ experiences related to the preoperative hypnosis.

*Participants*: Five participants were considered sufficient to achieve high information power, based on (1) the aim of the study, (2) its theoretical background, (3) the specificity of the sample, (4) the quality of the dialog, and (5) the strategy for analysis.^22^

*Inclusion*: All 5 participants fulfilled the inclusion-criteria: Female gender, 18 to 65 years of age, scheduled for breast cancer surgery at OUH, residents of the Oslo region, and sufficient Norwegian language skills.

*Recruitment*: Women scheduled for mastectomy or breast-conserving surgery were contacted over telephone by a nurse at the hospital. The nurse informed about the study, and those who expressed interest in participating, were then contacted by one of the authors (SER) who explained in detail what participation would entail.

Eleven women were invited to participate in this study, of which 5 agreed and 6 declined. Participation was voluntary, and there were no offers of financial incentives.

*Procedure*: The intervention was conducted 30 minutes prior to the participants’ scheduled operation (n = 4) or the day before surgery (n = 1). Prior to the hypnotic induction, the therapist (SER) explained the concept of hypnosis, and debunked common misunderstandings. Participants were given the chance to ask questions and address concerns. Finally, they were asked to answer a short questionnaire, assessing current pain intensity, discomfort, and anxiety related to the upcoming surgery, as well as expectations about pain after surgery. Subsequently, clinical hypnosis was conducted, and the participants were debriefed.

*Intervention*: The hypnosis script applied in this study was developed by a team at Mount Sinai School of Medicine in New York.^5^ A full version of the script is available in Hypnosis for Chronic Pain Management: Therapist Guide.^23^ For the purpose of this and further studies, the script was translated professionally and adjusted to a Norwegian context through multiple discussions and revisions in an appointed group of experts (SER, OAS, and NOC). The hypnosis intervention takes approximately 20 minutes to complete and consists of hypnotic induction, deepening of the hypnotic experience, suggestions, and conclusion of hypnosis. Additionally, the intervention includes instructions of how to perform self-hypnosis and encouragement to do so before and after the surgery.

*Interviews*: Semi-structured interviews were conducted 7 (n = 4) or 8 (n = 1) days after surgery, either face to face (n = 3) or over telephone (n = 2). The interviews were based on a standardized interview guide, which secured central topics to be addressed:

(1) How did you experience the period of time from you were diagnosed with breast cancer until surgery?
  - In this period, did you find anything particularly difficult or helpful?
  - What were your thoughts and feelings related to the upcoming surgery?
(2) How have you experienced the period of time after surgery?
  - Has anything been particularly challenging, difficult, or demanding?
  - Has anything been helpful?
(3) How did you experience the hypnosis intervention, with regards to how you felt after surgery?
(4) What do you think about hypnosis as a method to reduce pain and discomfort after surgery?
  - Do you believe it could be helpful in other surgical procedures?
(5) Did you find anything in the hypnosis intervention strange/weird, particularly useful, or were there anything you reacted to in particular?
  - Did you experience the hypnosis intervention as unnecessary or burdensome?
(6) Have you used self-hypnosis yourself after surgery?
  - How was it?/How come you did not use it?
(7) Prior to the hypnosis session, what were your beliefs about hypnosis, and have those beliefs changed?
(8) Was there anything in the session that you would have liked to be different?
(9) Is there anything you would like to add?

The interviewer (SBL) informed the participants that the study examined both positive and negative experiences, and the interviews were conducted in a way that allowed for disruptions in their structure. Consequently, the participants could pause or stop the interview at any time, go beyond the topics in the interview guide, ask questions to the interviewer and talk uninterruptedly about their experiences. The interviewer asked follow-up questions where appropriate. The interviews were audio-recorded and transcribed verbatim. The duration of the interviews varied from 8 to 25 minutes (average = 20.2, SD = 6.9, median = 22).

*Analysis*: The transcribed interviews were analyzed qualitatively, through systematic text condensation.^24^ Two of the authors (SBL and SER) coded independently and agreed upon 4 codes and 14 subgroups, which summarized the contents of the interviews.

*Ethical approval and data protection*: The study was presented and approved by the regional committee for medical and health research ethics (reference number: 2018/781) and advised by the Data Protection Officer at OUH (reference number: 18/06503). Written informed consent was obtained prior to the hypnosis session, after the participants had been given information about the study and their rights. All data were securely stored.

## Results

### The Survey from the General Population

The nation-wide and demographically balanced internet survey included a sample of 1049 adults (540 females, 509 males) representative of the Norwegian population. The sample had an average age of 47.5 years, which ranged from 18 to 93 years.

The results showed that 8.2% (n = 86) of the participants reported having tried hypnosis, while 0.6% (n = 6) indicated that they did not know whether they had tried hypnosis or not. When asked whether they would have said yes to hypnosis carried out by a health care professional, 14.1% (n = 148) responded “Very likely,” 24.8% (n = 260) responded “Pretty likely,” 28.5% (n = 299) responded “A bit likely,” 22.8% (n = 239) responded “Not likely at all,” and 9.8% (n = 103) responded “Do not know.” Thus, 67.4% (n = 707) reported some degree of willingness to accept hypnosis as part of medical care.

Table 1 illustrates the distribution and characteristics of the respondents who had previously tried hypnosis and those who were willing to accept hypnosis in a medical setting. Marginally more women than men had tried hypnosis.

**Table 1.** Sample Demographics of Respondents Categorized on How They Answered 2 Questions About Use and Attitudes Toward Hypnosis. Question 1 (Q1) “Have You Ever Tried Hypnosis?” Is Presented for Those Answering “Yes” or “No.” Question 2 (Q2) “How Likely is It That You Would Have Said Yes to Hypnosis Carried Out by Health Care Professional?” Was Dichotomized Into “Likely” (“Very Likely,” “Pretty Likely,” or “A Bit Likely”) and “Not Likely” (“Not Likely at All”) To Illustrate Differences on Demographics.

	Q1 yes	Q1 no	Q2 likely	Q2 not likely
	N (%)	N (%)	N (%)	N (%)
Gender				
Male	39 (7.7)	466 (92.3)	334 (73.6)	120 (26.4)
Female	47 (8.7)	491 (91.3)	373 (75.8)	119 (24.2)
Age (y)				
18-29	21 (10.6)	177 (89.4)	145 (80.1)	36 (19.9)
30-39	18 (9.7)	168 (90.3)	126 (73.7)	45 (26.3)
40-49	18 (9.3)	176 (90.7)	139 (81.3)	32 (18.7)
50-59	12 (6.6)	169 (93.4)	129 (77.7)	37 (22.3)
60-69	8 (5.6)	136 (94.4)	87 (68)	41 (32)
70-79	9 (7.3)	115 (92.7)	72 (63.7)	41 (36.3)
80-93	0 (0)	16 (100)	9 (56.3)	7 (43.7)
Education				
Primary	1 (5.9)	16 (94.1)	10 (66.7)	5 (33.3)
Lower secondary	8 (11.9)	59 (88.1)	48 (82.8)	10 (17.2)
Upper secondary	30 (8.5)	324 (91.5)	240 (76.4)	74 (23.6)
College/university (<4 y)	33 (8.8)	344 (91.2)	265 (76.1)	83 (23.9)
College/university (>4 y)	14 (6.1)	214 (93.9)	144 (68.2)	67 (31.8)
Income				
<300 000 NOK	12 (11.3)	94 (88.7)	73 (74.5)	25 (25.5)
300 000-499 999 NOK	13 (7.9)	152 (92.1)	114 (75.5)	37 (24.5)
500 000-799 999 NOK	20 (8.4)	218 (91.6)	156 (71.9)	61 (28.1)
800 000-999 999 NOK	16 (10.3)	139 (89.7)	115 (78.8)	31 (21.2)
1 000 000-1.5 M NOK	11 (6.5)	158 (93.5)	126 (78.8)	34 (21.2)
>1.5 M NOK	3 (6.7)	42 (93.3)	31 (77.5)	9 (22.5)
Not given	11 (6.7)	154 (93.3)	92 (68.7)	42 (31.3)
Region				
Eastern Norway	56 (9.6)	526 (90.4)	400 (76.2)	125 (23.8)
Southern Norway	4 (6.5)	58 (93.5)	49 (83.1)	10 (16.9)
Western Norway	13 (6.2)	197 (93.8)	133 (69.3)	59 (30.7)
Central Norway	11 (12)	81 (88)	62 (74.7)	21 (25.3)
Northern Norway	2 (2.1)	95 (97.9)	63 (72.4)	24 (27.6)

The correlation matrix showed that younger age (r = −.076, *P* < .05) was significantly correlated with previous hypnosis experience. This was not the case for any of the other sociodemographic characteristics (gender, educational level, number of people in household, income, and living in urban or rural areas).

The following demographic descriptors were correlated with willingness to accept hypnosis in a medical setting: Younger age (r = −.119, *P* < .001), lower educational level (r = −.065, *P* < .05), and not living alone (Number of people in household, r = .117, *P* < .001). The variables were coded the same way as in Table 1.

Finally, previous experience with hypnosis was also associated with willingness to try hypnosis (r = .104, *P* < .001).

### The Pilot Study

Of the 5 women included in the pilot study, 1 was 40 to 50 years old, 2 were between the age of 50 and 60, and 2 were 60 to 70 years old. See Table 2 for clinical characteristics of the sample.

**Table 2.** Participant Characteristics (N = 5) Before and After Surgery.

	ID:01	ID:02	ID:03	ID:04	ID:05
Breast cancer surgery	B	B	B	M	M
Hypnosis intervention, time	X	Y	X	X	X
Interview, days after surgery	7	7	7	8	7
Interview, face to face/telephone	F2F	F2F	F2F	Tel	Tel
NRS (0-10), preoperative pain	0	2	-	0	0
NRS (0-100), preoperative anxiety	0	30	60	30	10
NRS (0-100), expected postoperative pain	10	50	60	70	40
Expected need for postoperative pain medication (0-5)	1	4	3	3	2
NRS (0-10) postoperative pain, time of interview	0	0	2	2	1
NRS (0-10) postoperative fatigue, time of interview	0	3	3	2	3

Abbreviations: B, breast-conserving surgery; M, mastectomy; X, 30 min prior to surgery; Y, the day before surgery; F2F, face to face; Tel = telephone; NRS, numeric rating scale.

Through systematic text condensation, 4 codes (themes) and 14 subgroups were identified (Table 3), which summarized the contents of the interviews.

**Table 3.** Codes and Subgroups.

Codes	Subgroups
Caretaking	Positive caretaking and social support Negative caretaking and social support Caretaking of others
Experiences with hypnosis	Beliefs about hypnosis Experiences related to the hypnosis session Experiences related to self-hypnosis Perceived effect of hypnosis Experiences related to similar techniques Suggestions for improvement of the hypnosis intervention and session
The breast cancer diagnosis	Thoughts and feelings related to the diagnosis Coping strategies
The breast cancer surgery	Thoughts and feelings related to the surgery Positive postoperative reactions Negative postoperative reactions

1. Caretaking

The participants’ statements about physical and psychological caretaking were included in this code. These statements involved their experiences related to caretaking by health care professionals or their social environment, as well as their caretaking of others, both prior to and after surgery.

All the participants reported positive experiences related to caretaking and support, either from health care professionals at the hospital, or from their social environment: “*I have been confident in the health care professionals who have treated me”; “The house looked as though I had already died; there were flowers from here to eternity*” (ID:04). However, support could also be challenging, and sometimes provoke strong emotions: “*Meeting people who is saying ‘I know so many who have had breast cancer, and it has gone so well’, those people I just felt I wanted to punch*” (ID:04).

Many of the participants explained how the hypnosis intervention had given them an experience of being taken care of: “*I thought it was nice being seen as an individual and not just a number in the line that day*” (ID:03); “*The way you met me [during the hypnosis session] was very nice*” (ID:04); “*I would not have let just anyone do [the hypnosis], but I trusted you*” (ID:01).

One participant (ID:05) did not feel adequately taken care of at the hospital. She experienced a lack of coordination and had been misinformed about where and when to meet on the day of her surgery, leading to unnecessary long waiting. She further explained that the postoperative care was lacking as well, and she had not been given analgesics or a postoperative follow-up by the time of the interview.

Lastly, two of the participants described how the responsibility of taking care of their families were challenging during this period. One disclosed on her children’s fear of her dying, the need they had for consolation, and how this had been difficult to provide due to her own thoughts and feelings related to her diagnosis: “*It becomes a sort of mortal dread, especially for the children. There is a good chance of survival, but all [the children] can hear is that I have cancer and that they might lose me*” (ID:04).

2. Experiences with hypnosis

This code includes the participants’ beliefs about hypnosis, their experiences and the perceived effect of the given hypnosis, any suggestions for improvements, and their experiences with similar relaxation techniques.

The participants described how their beliefs about hypnosis were influenced by what they had seen on television and in the media. As such, many reported of a general skepticism prior to the hypnosis session: “*The general belief about hypnosis is that you will disappear completely and be in a trance where you are not conscious*” (ID:02); “*You have the idea that you will be completely gone during hypnosis, and that people can do all sorts of things, like you have seen on TV*” (ID:03). However, many of the participants reported that their skepticism was reduced when they received more information and underwent the hypnosis: “*It is the knowledge that has given me another belief about hypnosis than I used to have*” (ID:02); “*I suppose I am skeptical, . . . but I have to admit I was positively surprised*” (ID:03). Nevertheless, not all were skeptical to begin with: “*I think that [preoperative hypnosis] is really smart*”; “*I think you can use the cognitive to find a sort of tranquility in your body*” (ID:04).

Furthermore, many of the participants also had experiences with similar relaxation techniques—like mindfulness, meditation and yoga—which contributed to reduce their skepticism about hypnosis. These participants found similarities between hypnosis and the techniques they had experiences with, and they used these experiences to come deeper into the hypnotic state.

All the participants reported that hypnosis had been a positive experience: “*I found it delightful and relaxing*” (ID:05); “*It sort of became a more pleasant way into the surgery*” (ID:02). Consistently, none of the participants reported the hypnosis session as bothersome or distressing: “*There was absolutely nothing negative about [the hypnosis session]” (ID:03). However, many reported uncertainties about whether they had gotten deep enough into the hypnotic experience, both during the guided hypnosis session and during self-hypnosis: “I am not sure I got deep enough into hypnosis*” (ID:02); “I *imagine that I do not achieve the complete focus [during self-hypnosis]*” (ID:03).

All the participants commented on the perceived effect of hypnosis, and they all described a positive effect: “*For me it has worked very well*” (ID:01). Still, some pointed out that they could not know how their experiences would have been without the hypnosis: “*I do not know if [hypnosis] is the reason I am feeling so well, but it is surprising to experience the energy that has been gone for so long*” (ID:02).

In accordance with these descriptions, the participants reported a general satisfaction with the practical procedures and aspects of the intervention. When prompted, they did however share some suggestions for improvement. One suggestion mentioned by several of the participants was about being able to lie down, rather than sitting upright, during the session. One participant (ID:02) mentioned that having a blanket covering her during the session made her feel safe: “*When you lie there without [anything covering you], you feel vulnerable*.” Moreover, the majority of the participants expressed a wish to participate in more guided sessions, as well as having the opportunity to do the hypnosis earlier, preferably in the weeks before surgery: “*I could have needed this in the waiting period as well*” (ID:03). One participant (ID:05) pointed out the need for good coordination and communication between the therapist and the health care professionals working at the department. She explained how this would improve her experience as a patient. Others also highlighted the importance of information, especially regarding the practical implementation of the hypnosis.

3. We also inquired about the participants’ thoughts around the word “hypnosis,” and whether it was too stigmatized to be used in a Norwegian context. None of the participants felt the word “hypnosis” was too problematic, and one said: “*I think people best get used to it, because there will be a lot more of it in the future*” (ID:04). The breast cancer diagnosis

This code is about the participants’ thoughts and feelings related to their diagnosis, strategies of coping prior to and after surgery, and worries about the future.

Several of the participants described feelings of concern and powerlessness related to the diagnosis, as well as a general insecurity about the future. This included fears of not coming back to work, having a relapse, or dying: “*All those who get cancer have looked death in the eye*” (ID:05); “*[I have had] a lot of thoughts about changing the way I live. I have always worked a great deal, and perhaps I should think about living a bit differently once I am cancer free*”; “[I wonder] if I can ever be bright enough to work again. I feel like my head is not working properly. And when I think about that, I wonder if the cancer has spread to my head as well” (ID:04).

4. Despite the insecurity and fear, there were also reports of optimism about the future. One participant (ID:02) described it the following way: “*I am very optimistic and positive that this will all go well. At least I am counting on that, and if something else happens, I will deal with it then*”. Many also developed strategies of coping to deal with their thoughts and feelings related to their diagnosis, such as physical exercise (ID:05) and cognitive reappraisal (ID:04). The breast cancer surgery

The participants’ statements about their thoughts or feelings related to surgery, or their postoperative condition are included in this code.

Several participants experienced conflicting thoughts and feelings related to their surgery. Most dreaded it, but they also looked forward to being done with it since they recognized its necessity: “*All dread an operation like this*” (ID:05*);* “*The surgery is necessary if you are going to have any hope for recovery*” (ID:03). Furthermore, several participants were eager to start the treatment: “*You are kind of ready to get started*” (ID:04); “*I would very much like to get the [surgery] done and over with*” (ID:05).

All the participants reported that the time after surgery had been positive and associated with relief: “*After the surgery I felt like I had gotten more energy, and [I feel] a sort of relief of having removed [the tumor]” (ID:02); “I felt calm after [the surgery, and I thought that] at least that tumor could not do more damage*” (ID:03). However, several of the participants also reported of minor ailments, like dizziness, swelling, pain, emotional lability and fatigue.

## Discussion

The aim of this mixed-method study was to assess the feasibility of preoperative hypnosis among women undergoing breast cancer surgery in Norway. In order to do so, we conducted a representative survey in the general population to assess the experience with hypnosis in general, as well as willingness to accept hypnosis in a medical setting, and a qualitative pilot study investigating 5 patients’ experiences with preoperative hypnosis.

The evaluation of feasibility involved an assessment of acceptability of the intervention, response to the intervention, recruitment capacity, and data collection procedures.^21^ We use data from both the representative survey and the pilot study to inform the feasibility assessment.

### Acceptability

Attitudes toward hypnosis influence people’s interest in using hypnosis.^25^ The representative survey gives insight into the Norwegian population’s attitudes toward hypnosis. In the survey, as much as 67% expressed willingness to accept hypnosis in a medical setting. These results are in line with previous studies, where less than a third reject hypnotherapy or have negative attitudes toward clinical hypnosis.^9,11^ In our survey, some demographic variables (younger age, lower educational level, and not living alone) were weakly but significantly correlated with greater willingness to try hypnosis. Previous research regarding demographic variables show inconsistent findings. Montgomery et al^25^ found that only gender influences interest in hypnosis, whereas Krouwel et al^9^ report that younger age has been shown to impact willingness to try hypnosis. Nevertheless, both our survey and previous studies find that past experiences with hypnosis have an impact on interest in using hypnosis.^10,25^ The pilot study can provide some insight into why attitudes vary. Several of our participants described skepticism prior to recruitment, saying that their attitudes were influenced by hypnosis used for the purpose of entertainment. Still, the participants in our study had all agreed to participate and consequently been willing to accept hypnosis. This could mean that our pilot participants had more positive attitudes toward hypnosis than the general population, or that they were less influenced by their initial attitudes and therefore open to try hypnosis despite their skepticism.

Moreover, the participants’ positive attitudes are evident in the high adherence rate. Though some of the participants in the pilot study had difficulties with following all the suggestions, none of them expressed unwillingness to follow suggestions or instructions made by the therapist. This highlights the necessity of a collaborative relationship between the patient and the therapist.^9,26^ Furthermore, all the participants had used self-hypnosis prior to or after surgery, further improving the adherence-rate. Thus, the participants all accepted the study procedure and the intervention and reported that the hypnosis had been a positive experience. None experienced the hypnosis as bothersome or burdensome, though some suggestions for improvements were made, including a preference for receiving the hypnosis while lying down, the possibility of receiving multiple sessions, preference for a blanket during the session, and the need for good coordination between the therapist and the surgical department.

Previous experience with hypnosis improves attitudes toward hypnosis, and some of these changes in attitudes are even associated with better treatment outcomes.^26^ This was supported in our population survey where previous experience with hypnosis was associated with a higher willingness to accept hypnosis as part of medical care. The same appeared in the pilot study, where some reported that their attitudes toward hypnosis had changed to the better after receiving hypnosis. In addition, some of the participants reported that previous experiences with similar techniques, such as mindfulness and meditation, helped reduce their initial skepticism, as hypnosis reminded them of those experiences.

The information provided by the research team could also have had an impact on the attitudes toward hypnosis. In the survey, the respondents were told that hypnosis can be useful in medical treatment. It is unclear whether this information impacted the responses. In the pilot study, the participants were provided information about the study and the intervention on multiple occasions, and this information was reported to improve the participants’ attitudes. Many of the participants reported that both the phone call from the therapist (SER) and the debunking prior to the hypnosis session, helped them gain a better understanding of what hypnosis is (and is not). This might have had an impact on the appeal and interest of the intervention,^25^ reassuring the participants that they remain in control and conscious during the whole procedure.^9^ The information might also have changed the participants’ expectancies of their hypnotizability.^27^ Studies have shown that these expectancies correlate with actual hypnotizability, which—as well as hypnotic responsiveness and hypnotic susceptibility—is associated with attitudes toward hypnosis.^26,27^ Furthermore, the participants were informed that there are no known adverse side-effects to this treatment, and according to some of the participants this had an impact on their willingness to try hypnosis. They were also informed that the intervention would be performed by a trained psychologist associated with the medical establishment, which most people generally prefer.^9^

The survey results confirm and complement the findings in the clinical pilot study in that despite a widespread skepticism toward hypnosis, there is nevertheless a willingness to try, and possibly more positive attitudes after having experienced hypnosis or been provided with adequate information.

### Recruitment Capacity

While 67% in the survey reported a willingness to accept hypnosis in a medical setting, only 5 out of 11 (45%) of the patients who were asked to participate in the pilot study accepted. This relatively low response rate could therefore depend on factors other than willingness, such as the timing and content of the first introduction: The time of recruitment happened shortly after the women had been diagnosed with breast cancer, and the invitation was provided by health care staff with limited knowledge about hypnosis.

Despite the modest recruitment rate, we experienced no difficulties with the recruitment criteria. Thus, the recruitment criteria do not seem to be related to the recruitment rate, and other factors may be more relevant to consider: First, some reported that this study would be too much to consider in an otherwise overwhelming situation. Many breast cancer patients experience high levels of stress and worry prior to their surgery,^28^ which could influence the women’s willingness to participate in a research study. Second, attitudes toward hypnosis could also have influenced the recruitment process. Several of the participants reported that their beliefs about hypnosis were affected by what they had seen in the mainstream media, which was dominated by mysteries and myths. The women who declined the invitation could as such have been more skeptical toward hypnosis, or more affected by their preliminary attitudes, which may have influenced their willingness to try hypnosis.^25^

Furthermore, the sample characteristics were as expected. Two of the most common types of surgery were represented, including 3 of 5 patients receiving breast conserving surgery which is just below the national rates of 81%.^29^

Based on these preliminary findings, we do not anticipate major problems in recruiting participants for a large-scale trial, but it seems reasonable to expect a response rate of about 50%.

### Data Collection Procedures

Although the pilot study is not a small-scale version of a randomized controlled trial, the data collection procedures in the pilot study can still inform further studies.

In the pilot study, the participants were asked to rate their level of pain, anxiety and pain expectancy prior to surgery. They were also asked to rate their level of postoperative pain and fatigue at the time of the interview. Both prior to and after surgery, their ratings were done on the Numeric Rating Scale (0-10 or 0-100), which is a reliable and valid measure.^30,31^ None of the participants had difficulty reporting on this measure, though some commented that it was easier to use the 0 to 10 rating than the 0 to 100 rating.

Getting in touch with the participants after surgery and conducting the interview also proceeded without any encountered problems. The participants were all able to give an account of their experiences regarding hypnosis and the study procedure at the time of the interview.

Thus, collecting data prior to and 1 week after surgery was feasible in the pilot study, which implies that it should be feasible in the large-scale trial as well.

### Responses and Experiences

The main objective of a feasibility study is not to test efficacy.^20^ Nevertheless, our pilot results do provide some indications of effect, as experienced and attributed by our 5 participants. All 5 participants reported that the hypnosis had been a positive and/or pleasant experience, and no adverse or negative side effects were described. This is consistent with results from numerous previous trials where no adverse effects have been reported from hypnosis in surgical settings.^6,32^ Moreover, none of the 5 participants reported moderate or severe pain 1 week after surgery, which is relatively unusual after breast cancer surgery,^33^ and all of the participants experienced less pain after surgery than what they expected prior to surgery. Furthermore, the results of the pilot study align well with the previous US studies of preoperative hypnosis as an effective method to reduce pain and other side effects from breast cancer surgery.^5,6^

Thus far, the study with the most promising effects is a randomized controlled trial from the US.^5^ In that trial, a short preoperative hypnosis had an impact on the patients’ need for pain medications and the level of postoperative pain, nausea, exhaustion, and discomfort, with moderate to large effect sizes. In a French study, following a similar protocol, but with some deviations from the American study, the effect of preoperative hypnosis was not reproduced.^34^ As such there is a need for further replications in different contexts.

### Implications for Future Studies

The results from this feasibility study have several implications. First, it is essential that participants are given adequate information about hypnosis in a medical context. Debunking common misunderstandings is important, as they will likely impact attitudes toward participating. Providing proper information, both during and after recruitment, could improve attitudes toward hypnosis and possibly have an impact on engagement and adherence.^25^ Second, the feasibility study prepares us for a moderate recruitment rate, and in further studies it would be of importance to investigate how information prior to recruitment could be optimized to reduce negative attitudes and as such improve recruitment rates. Third, it is important that the hypnosis and the logistics related to it are not perceived as additional stressors to the participants. A dynamic collaboration between the therapist and the surgical department is essential. This could for instance involve securing the therapist access to important channels of clinical information (eg, medical records, email lists, etc.). Finally, the participants’ specific recommendations reported here give us insight into the participants’ needs and wishes, both prior to, during and after the hypnosis session. These recommendations should be considered in future trials in order to improve the patient experience.

### Strengths and Limitations

The strengths of this study include the mixed-method design. The quantitative survey provides representative data with high generalizability, while the pilot study provides rich data with more depth and transferability. Furthermore, the intervention did not interfere with the standard course of treatment but was integrated as a complementary treatment. This strengthens feasibility as it does not deprive patients of any aspects of the standard treatment, and only impose a minimal burden.

The limitations include the small and selected sample in the qualitative pilot study, which likely excluded more skeptical participants and their experiences. Nevertheless, as the aim was to assess feasibility of hypnosis in this setting, the experiences of the consenting participants were most relevant in this regard. In line with this, our research group recently initiated a large-scale study where the effect of preoperative hypnosis will be assessed in a randomized controlled trial of 200 women undergoing breast cancer surgery.

## Conclusion

The results from this mixed-method feasibility study indicate an openness to hypnosis in a medical setting, both in the general population and in patients with breast cancer. The overall results indicate mostly positive attitudes and willingness to accept hypnosis as part of medical care, as indicated in both the representative survey and the qualitative reports. The practical procedure of the intervention was feasible, without obstacles, and the participants’ experiences of the preoperative hypnosis were positive. There were no reports of adverse side effects from the hypnosis, and none of the participants experienced more than mild pain intensity 1 week after surgery, which strengthens the hypothesis of an analgetic effect of hypnosis in this setting. Preoperative hypnosis thus appears feasible as a complementary treatment for women scheduled for breast cancer surgery in Norway.

## References

[1] Kehlet H Jensen TS Woolf CJ. Persistent postsurgical pain: risk factors and prevention. Lancet. 2006;367:1618-1625. doi:10.1016/S0140-6736(06)68700-X 16698416

[2] Niraj G Rowbotham DJ. Persistent postoperative pain: where are we now? Br J Anaesth. 2011;107:25-29. doi:10.1093/bja/aer116 21610014

[3] *Kreftregisteret Årsrapport 2018 med resultater og forbedringstiltak fra Nasjonalt kvalitetsregister for brystkreft* . Nasjonalt kvalitetsregister for brystkreft; 2019. https://www.kreftregisteret.no/globalassets/publikasjoner-og-rapporter/arsrapporter/publisert-2019/arsrapport-2018-brystkreft.pdf. Accessed June 20, 2021.

[4] McPherson K Steel CM Dixon JM. ABC of breast diseases. Breast cancer-epidemiology, risk factors, and genetics. BMJ. 2000;321:624-628. doi:10.1136/bmj.321.7261.624 10977847 PMC1118507

[5] Montgomery GH Bovbjerg DH Schnur JB , et al. A randomized clinical trial of a brief hypnosis intervention to control side effects in breast surgery patients. J Natl Cancer Inst. 2007;99:1304-1312. doi:10.1093/jnci/djm106 17728216

[6] Montgomery GH David D Winkel G , et al. The effectiveness of adjunctive hypnosis with surgical patients: a meta-analysis. Anesth Analg. 2002;94:1639-1645, table of contents. doi:10.1097/00000539-200206000-00052 12032044

[7] Montgomery GH Weltz CR Seltz M , et al. Brief presurgery hypnosis reduces distress and pain in excisional breast biopsy patients. Int J Clin Exp Hypn. 2002;50:17-32. doi:10.1080/00207140208410088 11778705

[8] Kekecs Z Nagy T Varga K. The effectiveness of suggestive techniques in reducing postoperative side effects: a meta-analysis of randomized controlled trials. Anesth Analg. 2014;119:1407-1419. doi:10.1213/ANE.0000000000000466 25289661

[9] Krouwel M Jolly K Greenfield S. What the public think about hypnosis and hypnotherapy: a narrative review of literature covering opinions and attitudes of the general public 1996-2016. Complement Ther Med. 2017;32:75-84. doi:10.1016/j.ctim.2017.04.002 28619308

[10] Molina-Peral JA Rodríguez JS Capafons A , et al. Attitudes toward hypnosis based on source of information and experience with hypnosis. Am J Clin Hypn. 2020;62:282-297. doi:10.1080/00029157.2019.1584741 31928518

[11] Palsson O Twist S Walker M. A national survey of clinical hypnosis views and experiences of the adult population in the United States. Int J Clin Exp Hypn. 2019;67:428-448. doi:10.1080/00207144.2019.1649538 31526263

[12] Salomonsen LJ Grimsgaard S Fønnebø V. Bruk av alternativmedisinsk behandling ved norske sykehus. Tidsskr Nor Laegeforen. 2003;123:631-633. https://tidsskriftet.no/2003/03/aktuelt/bruk-av-alternativmedisinsk-behandling-ved-norske-sykehus. Accessed June 20, 2021. 12683190

[13] Salomonsen LJ Skovgaard L la Cour S , et al. Use of complementary and alternative medicine at Norwegian and Danish hospitals. BMC Complement Altern Med. 2011;11:4. doi:10.1186/1472-6882-11-4 21244655 PMC3033860

[14] Jacobsen R Fønnebø VM Foss N , et al. Use of complementary and alternative medicine within Norwegian hospitals. BMC Complement Altern Med. 2015;15:275. doi:10.1186/s12906-015-0782-5 26268605 PMC4534010

[15] Bennett BM Laidlaw TM Dwivedi P , et al. A qualitative study of the experience of self-hypnosis or Johrei in metastatic breast cancer using interpretative phenomenological analysis. Contemp Hypn. 2006;23:127-140. doi:10.1002/ch.318

[16] Del Mar Hita Millan I Cameron J Yang Y , et al. An observational mixed-methods approach to investigate the fear of cancer recurrence cognitive and emotional model by Lee-Jones et al with women with breast cancer during radiotherapy treatment. Ecancermedicalscience. 2019;13:984. doi:10.3332/ecancer.2019.984 32010208 PMC6974375

[17] Denieffe S Gooney M. A meta-synthesis of women’s symptoms experience and breast cancer. Eur J Cancer Care. 2010;20:424-435. doi:10.1111/j.1365-2354.2010.01223.x 20825463

[18] Liu L Wu Y Cong W , et al. Experience of women with breast cancer undergoing chemotherapy: a systematic review of qualitative research. Qual Life Res. 2021;30:1249-1265. doi:10.1007/s11136-020-02754-5 33459972

[19] Montazeri A. Health-related quality of life in breast cancer patients: a bibliographic review of the literature from 1974 to 2007. J Exp Clin Cancer Res. 2008;27:32. doi:10.1186/1756-9966-27-32 18759983 PMC2543010

[20] Lancaster GA. Pilot and feasibility studies come of age! Pilot Feasibility Stud. 2015;1:1. doi:10.1186/2055-5784-1-1 29611687 PMC5842886

[21] Orsmond GI Cohn ES. The distinctive features of a feasibility study: objectives and guiding questions. OTJR (Thorofare N J). 2015;35:169-177. doi:10.1177/1539449215578649. 26594739

[22] Malterud K Siersma VD Guassora AD. Sample size in qualitative interview studies: guided by information power. Qual Health Res. 2016;26:1753-1760. doi:10.1177/1049732315617444 26613970

[23] Jensen MP. Hypnosis for Chronic Pain Management: Therapist Guide. Oxford University Press; 2015.

[24] Malterud K. Systematic text condensation: a strategy for qualitative analysis. Scand J Public Health. 2012;40:795-805. doi:10.1177/1403494812465030 23221918

[25] Montgomery GH Sucala M Dillon MJ , et al. Interest and attitudes about hypnosis in a large community sample. Psychol Conscious (Wash D C). 2018;5:212-220. doi:10.1037/cns0000141 30035144 PMC6052866

[26] Mendoza ME Capafons A Jensen MP. Hypnosis attitudes: treatment effects and associations with symptoms in individuals with cancer. Am J Clin Hypn. 2017;60:50-67. doi:10.1080/00029157.2017.1300570 28557676

[27] Koep LL Biggs ML Rhodes JR , et al. Psychological mindedness, attitudes toward hypnosis, and expectancy as correlates of hypnotizability. Int J Clin Exp Hypn. 2020;68:68-79. doi:10.1080/00207144.2020.1682255 31914366

[28] Montgomery GH Schnur JB Erblich J , et al. Presurgery psychological factors predict pain, nausea, and fatigue one week after breast cancer surgery. J Pain Symptom Manag. 2010;39:1043-1052. doi:10.1016/j.jpainsymman.2009.11.318 PMC2918882 20538186

[29] Kreftregisteret. Årsrapport 2019 med resultater og forbedringstiltak fra Nasjonalt kvalitetsregister for brystkreft. Nasjonalt kvalitetsregister for brystkreft; 2020. https://www.kreftregisteret.no/globalassets/publikasjoner-og-rapporter/arsrapporter/publisert-2020/arsrapport-2019-nasjonalt-kvalitetsregister-for-brystkreft.pdf. Accessed June 20, 2021.

[30] Breivik H Borchgrevink PC Allen SM , et al. Assessment of pain. Br J Anaesth. 2008;101:17-24. doi:10.1093/bja/aen103 18487245

[31] Hawker GA Mian S Kendzerska T , et al. Measures of adult pain: visual analog scale for pain (VAS pain), numeric rating scale for pain (NRS pain), McGill pain questionnaire (MPQ), short-form McGill pain questionnaire (SF-MPQ), chronic pain grade scale (CPGS), short form-36 bodily pain scale (SF-36 BPS), and measure of intermittent and constant osteoarthritis pain (ICOAP). Arthritis Care Res. 2011;63 Suppl 11:S240-S252. doi:10.1002/acr.20543 22588748

[32] Nowak H Zech N Asmussen S , et al. Effect of therapeutic suggestions during general anaesthesia on postoperative pain and opioid use: multicentre randomised controlled trial. BMJ. 2020;371:m4284. doi:10.1136/bmj.m4284 PMC7726311 33303476

[33] Okamoto A Yamasaki M Yokota I , et al. Classification of acute pain trajectory after breast cancer surgery identifies patients at risk for persistent pain: a prospective observational study. J Pain Res. 2018;11:2197-2206. doi:10.2147/JPR.S171680 30323654 PMC6179582

[34] Amraoui J Pouliquen C Fraisse J , et al. Effects of a hypnosis session before general anesthesia on postoperative outcomes in patients who underwent minor breast cancer surgery: the HYPNOSEIN randomized clinical trial. JAMA Netw Open. 2018;1:e181164. doi:10.1001/jamanetworkopen.2018.1164 PMC6324272 30646110

